# Practical, epistemic and normative implications of algorithmic bias in healthcare artificial intelligence: a qualitative study of multidisciplinary expert perspectives

**DOI:** 10.1136/jme-2022-108850

**Published:** 2023-02-23

**Authors:** Yves Saint James Aquino, Stacy M. Carter, Nehmat Houssami, Annette Braunack-Mayer, Khin Than Win, Chris Degeling, Lei Wang, Wendy A Rogers

**Affiliations:** 1 Australian Centre for Health Engagement, Evidence and Values, School of Health and Society, University of Wollongong, Wollongong, New South Wales, Australia; 2 School of Public Health, The University of Sydney, Sydney, New South Wales, Australia; 3 The Daffodil Centre, Sydney, New South Wales, Australia; 4 Centre for Persuasive Technology and Society, Faculty of Engineering and Information Sciences, University of Wollongong, Wollongong, New South Wales, Australia; 5 Centre for Artificial Intelligence, School of Computing and Information Technology, University of Wollongong, Wollongong, New South Wales, Australia; 6 Department of Philosophy and School of Medicine, Macquarie University, Sydney, New South Wales, Australia

**Keywords:** Ethics, Decision Making, Policy, Information Technology

## Abstract

**Background:**

There is a growing concern about artificial intelligence (AI) applications in healthcare that can disadvantage already under-represented and marginalised groups (eg, based on gender or race).

**Objectives:**

Our objectives are to canvas the range of strategies stakeholders endorse in attempting to mitigate algorithmic bias, and to consider the ethical question of responsibility for algorithmic bias.

**Methodology:**

The study involves in-depth, semistructured interviews with healthcare workers, screening programme managers, consumer health representatives, regulators, data scientists and developers.

**Results:**

Findings reveal considerable divergent views on three key issues. First, views on whether bias is a problem in healthcare AI varied, with most participants agreeing bias is a problem (which we call the bias-critical view), a small number believing the opposite (the bias-denial view), and some arguing that the benefits of AI outweigh any harms or wrongs arising from the bias problem (the bias-apologist view). Second, there was a disagreement on the strategies to mitigate bias, and who is responsible for such strategies. Finally, there were divergent views on whether to include or exclude sociocultural identifiers (eg, race, ethnicity or gender-diverse identities) in the development of AI as a way to mitigate bias.

**Conclusion/significance:**

Based on the views of participants, we set out responses that stakeholders might pursue, including greater interdisciplinary collaboration, tailored stakeholder engagement activities, empirical studies to understand algorithmic bias and strategies to modify dominant approaches in AI development such as the use of participatory methods, and increased diversity and inclusion in research teams and research participant recruitment and selection.

## Background

 Artificial intelligence (AI) in healthcare has the potential to improve diagnostic accuracy, personalise therapeutic management and augment skills of healthcare workers. However, AI has also been subject to increasing ethical scrutiny.^1^,^4^ One dominant normative concern is the problem of bias: the effect of power asymmetries expressed in data and infrastructure.^5^,^7^ In a healthcare context, the presence of algorithmic bias means that without careful intervention, AI systems will disadvantage already under-represented and marginalised groups, systematically worsening existing inequity in healthcare systems.

In the context of AI, the term ‘bias’ is challenging as it has numerous connotations across disciplines. For statisticians, bias refers to a systematic error where the results do not reflect the true estimate. This kind of bias occurs for many reasons, including imbalance or unrepresentativeness of data.^8^ In the social sciences, bias broadly refers to systematic preferences, dispositions or inclinations in human thinking.^9^ Social bias, in particular, refers to problematic dispositions held by individuals or groups in favour of or against other individuals or groups based on numerous factors such as inappropriate generalisation (stereotyping) and inaccurate information, among others.^10^ Some theorists identify forms of bias that are of interest to this paper: (A) prejudice as negative attitudes towards individuals or groups and (B) discrimination as practices that unfairly advantage or disadvantage individuals or groups.^11^ In healthcare, different forms of social biases lead to disparities in access, service provision and treatment outcomes, ultimately leading to health inequities among social groups, making bias in healthcare AI a particularly compelling issue.

‘Algorithmic bias’ consists of both statistical and social meanings as it refers to systematic errors in AI systems that lead to results, interpretations or recommendations that unfairly advantage or disadvantage certain individuals or groups.^12^ For example, a US-based classification algorithm for skin cancer diagnosis,^13^ which was trained using mainly images of skin lesions in light-skinned patients, has been found to have approximately half the diagnostic accuracy when used on images of lesions among African-American patients.^14^ Such inaccuracy could worsen disparities in skin cancer outcomes, given African-Americans have the highest mortality rate for melanoma.^14 15^ Similar problems of biased results have been demonstrated in AI systems used for radiography-based disease diagnosis^15^ and health resource allocations.^16^ The statistical and social causes of algorithmic bias has been discussed elsewhere,^17^ but what these examples demonstrate is the potential of biased AI systems to not only replicate but amplify existing health inequities.^1^

The growing evidence of bias in healthcare AI systems has motivated various solutions, as well as critiques of these solutions, to mitigate algorithmic bias. These range from broad calls for ‘open science’ that promotes principles of ‘inclusivity, openness and trust’^14^ to specific evaluation tools such as the Prediction model Risk Of Bias ASsessment Tool.^18^ These solutions are yet to be widely implemented. Of particular, challenge is the inability of AI systems to accurately record and incorporate social data, which include cultural beliefs, economic status, linguistic identity and other social determinants of health.^19^ While efforts to address algorithmic bias are increasingly interdisciplinary, some scholars argue that the dominant approach of relying on data science mechanisms alone fails to address structural and systemic causes of marginalisation.^20^,^22^ Feminist ethicopolitical critiques highlight factors including historically entrenched power structures,^22^ exclusionary social demographics in the AI workforce,^20 21^ and sociocultural legacies of colonialism^23^ as some of the key drivers of algorithmic bias that deserve attention.

Being biased or prejudiced is an epistemic issue: it is a fault in one’s knowing about others. Several decades of psychological research has demonstrated that this fault is also often implicit. Humans tend to have prejudices against stigmatised groups of which they are unaware: what Fricker calls ‘the attitudinal fall-out from a semitoxic social environment’.^24^ When algorithmic systems absorb and reproduce this widespread implicit human bias, it can become automated and amplified; the human tendency to agree with machine decisions may then instantiate the epistemic error more firmly. Bias is also a moral issue: being prejudiced or biased may be morally blameworthy, and the existence of bias or prejudice raises questions of who should be responsible for addressing it. We will rely on Fricker’s view of responsibility for implicit prejudice to consider responsibility for bias in healthcare AI systems. On this view, we are rightly held responsible ‘not only for conduct based on things we know but also for conduct based on things we should have known but did not’, and not only for those things we can control, ‘but also those things we ought to be able to control’. Fricker shows that individuals may be non-culpable and yet responsible for the prejudiced thinking they may think themselves immune to, but in fact have absorbed from society. Fricker also shows that this may raise specific epistemic and moral obligations to put things right, which may include taking responsibility, making amends and changing procedures in institutions to prevent the same harms occurring again. These obligations may inhere at both individual and collective levels.

This analysis of responsibility for prejudicial errors in knowledge is highly relevant to our discussion. Despite the growing evidence on and proposed frameworks for addressing the problem of algorithmic bias, the moral and political seriousness of the problem remains contested within data science and AI communities. Those who create AI are overwhelmingly male and white,^7^ and this demographic skewing is worsening rather than improving. Many of those critiquing algorithmic bias are from minorities within the coding community, and often there is strong resistance from the ‘mainstream’ within their profession.^7^ Given this pattern of resistance, the question remains: Why does the problem of algorithmic bias persist despite growing efforts to counter it? And should stakeholders be held responsible for mitigating bias, even if they are not individually culpable for its cause?

Thus, in this paper we aim to: (1) understand whether AI and healthcare professionals see algorithmic bias in healthcare as a problem; (2) understand the range of strategies stakeholders endorse to attempt to mitigate algorithmic bias and (3) consider the ethical question of responsibility for algorithmic bias.

## Methodology

For purposes of recruitment, interview discussion and analysis, we used the Consolidated Standards of Reporting Trials–Artificial Intelligence's definitions of AI and machine learning (ML): AI broadly as interrelated technologies that can perform tasks normally requiring human intelligence; and ML as a set of approaches to AI that are designed to solve specific tasks by learning patterns from data, rather than by following explicit rules.^25^ We conducted qualitative, semistructured interviews with a diverse group of professionals involved in developing, selling, regulating or implementing healthcare AI, as further outlined below. Interviews were broad ranging, focusing on the ethical, legal and social implications of implementing AI in healthcare. One key issue discussed extensively by informants was algorithmic bias; the data from these discussions are the focus of this paper.

### Recruitment and sampling

We sought to recruit local and international participants. We aimed to access participants with specialist AI expertise and/or professional or clinical expertise; our inclusion criteria required that informants be involved in some way in the development, deployment and/or regulation of healthcare AI, and were at least knowledgeable enough to be informative about the potential implications of AI in their field. The sampling strategy was designed both to elicit diversity of views, and to allow comparisons between stakeholder groups. Initially, we recruited via an expression of interest to participate on Twitter and in newsletters and mailing lists of AI-related organisations. We also directly approached experts with relevant public profiles and invited them to participate. Over time, our sampling became more theoretically informed,^26^ and we invited experts who might help us address gaps in our analysis.

### Data collection

YSJA, a clinician and bioethicist trained in social science research methodologies, performed semistructured interviews via Zoom or telephone, taking between 20 and 90 min. The question guide covered views about healthcare AI development in Australia and internationally, imagined futures for healthcare AI, how AI might or might not change things for clinicians and service users, key ethical issues and how they should be addressed in practice, and AI regulation.^27^,^29^ Not all participants were asked all questions, either because they had limited time and we had recruited them to answer particular questions, or because they were recruited later in the study and we had already reached theoretical saturation for some categories.

### Data analysis

With participants’ consent, interviews were audiorecorded and professionally transcribed. Analysis was led by YSJA in collaboration with the research team. All participants were assigned a code that included their participant number and a summary of their roles; all transcripts were deidentified using these codes before analysis. Analysis combined approaches from constructivist grounded theory^26^ and the framework approach.^30 31^ The analysis steps were as follows: (1) coding interview transcripts; (2) memo-writing on each interview to develop an analytic understanding of how that informant strengthened the data on existing categories or added new categories to the analysis; (3) organising findings into a framework, including both analytical summaries and data excerpts and (4) memo-writing on each of the core concepts in the analysis. Codes and key themes were generated both deductively—that is, based on concepts from the bioethics and AI ethics literature^27 29 32^—and inductively.^28^ Data analysis was performed concurrently with data collection and data collection was modified to reflect insights from the developing analysis.

## Results

Saturation on core themes, including algorithmic bias, was reached after interviews with 72 participants. The majority of participants (n=54) worked in general diagnosis and screening, the rest were involved in breast cancer (n=10) and cardiovascular disease (CVD, n=8) diagnosis and screening, respectively (table 1). While most participants tended to have multiple forms of expertise, they could be classified based on self-identified primary roles, namely clinicians (n=22), regulators (n=17), developers/data scientists (n=10), researchers (n=8), healthcare administrators (n=5) and consumer representatives (n=3). We did not collect data on gender and racial identities of participants.

**Table 1:** Summary of participants’ expertise

Primary role	Examples	General	Breast	CVD	Total
Clinicians	Radiologists, GPs, emergency physicians, cardiologists, oncologists, imaging specialists	14	4	4	22
Health consumers	Consumer representatives	3	0	0	3
Developers	Computer scientists, health informaticians, software engineers	8	0	2	10
Entrepreneurs	medical officers in medical technology companies, start-up CEOs	7	0	0	7
Regulatory experts	Policy makers, policy officers, lawyers	16	1	0	17
Researcher	Academics or professional researchers outside the university sector	6	0	2	8
Healthcare administrators	Screening programme managers	0	5	0	5
		54	10	8	72

CEO, chief executive officer; CVD, cardiovascular disease; GP, general physician.

Our results show participants disagreed on three counts. First, participants disagreed whether or not algorithmic bias exists. Second, there was disagreement about whose responsibility it should be to implement strategies to mitigate bias. Participants who agreed that bias is a problem offered a variety of strategies to mitigate bias, ranging from sociolegal approaches to data science mechanisms. These mechanisms included calls for data governance, equitable research methods and increased diversity in datasets. Finally, for participants who suggested increasing diversity of or representativeness in datasets, there was disagreement on how to handle variables representing complex social information, such as gender and racial identities, that are typically marked with historical or continuing prejudice and marginalisation.

### Disagreement 1: views about whether algorithmic bias exists

The majority of participants took a *bias-critical view*. These experts were mainly clinicians and regulators, with a small number from other roles. These participants noted potential harms of algorithmic bias, ranging from medico-technical harms (eg, compromising patient safety) to broader social and public health harms (eg, perpetuating health inequity). Participants implied that such harms could undermine the promise of healthcare AI.

And …the researchers … discovered that, in this risk assessment, many essentially produced by the algorithm, there was a bias against African Americans. … if you are black American… you are certainly more likely to be mislabelled as low risk if you actually were high risk. Informant 22, regulatory expert

Some participants taking the bias-critical view also critiqued misconceptions about AI or computer systems that could lead people to dismiss the problem of algorithmic bias. The misconceptions include misguided faith in AI systems as objective and infallible.

For people who are less informed—have this view that kind of computers or machines or programs are sort of objective and they are black and white in their coding and so they are right or they are wrong and they won’t make mistakes, so to speak. I guess, my position on it is that some of the fallibilities that these systems have are based on the same problems that the human brain has in that it can only work with what’s been put into it. Informant 45, consumer representative

I think that there’s subjectivity in a lot of [AI] that we act like it’s very objective but it’s not. We’re making all kinds of assumptions in our machine learning, algorithms, in the default or the parameters that we set. Informant 56, researcher in bioinformatics

Two other views were expressed by participants. A small group of participants from all roles conveyed a *bias-apologist view*. On this view, bias was recognised as a problem to some extent, but not seen as an ethical concern—or at least, did not raise any more ethical concerns than the status quo. Some participants argued that bias in AI is unintentional. In contrast to the bias-critical view, the bias-apologist view emphasised the potential benefits of AI, despite the presence of bias. Participants expressing this view argued that bias was an ongoing problem even among clinicians—and AI bias was preferable to human bias.

Obviously an algorithm will have biases as a human has biases, but those biases are able to be measured. Informant 1, screening manager

First of all I’d say, what, and doctors aren’t biased? I’m sorry. Don’t get me started on doctor bias. We’re used to hurting people the old way, with biased old doctors. Now we’ve got a new set of biases, what are we going to do with them? But it is a valid point. Informant 55, entrepreneur

A third small group expressed a *bias-denial view*. These participants included developers, entrepreneurs and screening programme managers. No regulatory or legal expert subscribed to this view. Participants who expressed this view argued that bias existed in other industries but not medicine, or existed in other countries but not in Australia.

I don’t know that bias is one that we’ve seen any evidence that is being realised here [in Australia]. I think it’s more of a concern in health systems like the US. Informant 2, entrepreneur

Now imaging, you mentioned imaging? I don’t think there’s any good data or bad data in imaging. An image is just an image that you create from a machine, or like a CT scan or an x-ray. And then you feed that to the machine and it’ll detect and whatever it finds, it finds. Informant 52, developer

Some participants expressing the bias-denial view argued that bias was not likely to occur in medical diagnosis because imaging technologies are objective and the human body is universal, a direct contrast to the bias-critical view that claimed AI systems are not always objective or infallible.

I can only tell you that in radiology, if we do a C-T scan on your brain and on my brain, it will look exactly the same with a variation of black and white pixels. It doesn’t matter if you’re black, or white, or green, or blue, or whether you’re Australian or Chinese, a rib fracture is a rib fracture and the same is true for a vertebrae fracture or a pneumothorax. There is no bias there. Informant 59, clinician

Actually, I’d have no idea with the pathology samples what a person looks like so we don’t even know that. And I don’t know, you’d have to ask the doctor. I think the internal organs from people from different race, backgrounds, are probably pretty similar but I don’t know. Informant 50, developer

### Disagreement 2: views about acting to mitigate algorithmic injustice

**Figure 1:**
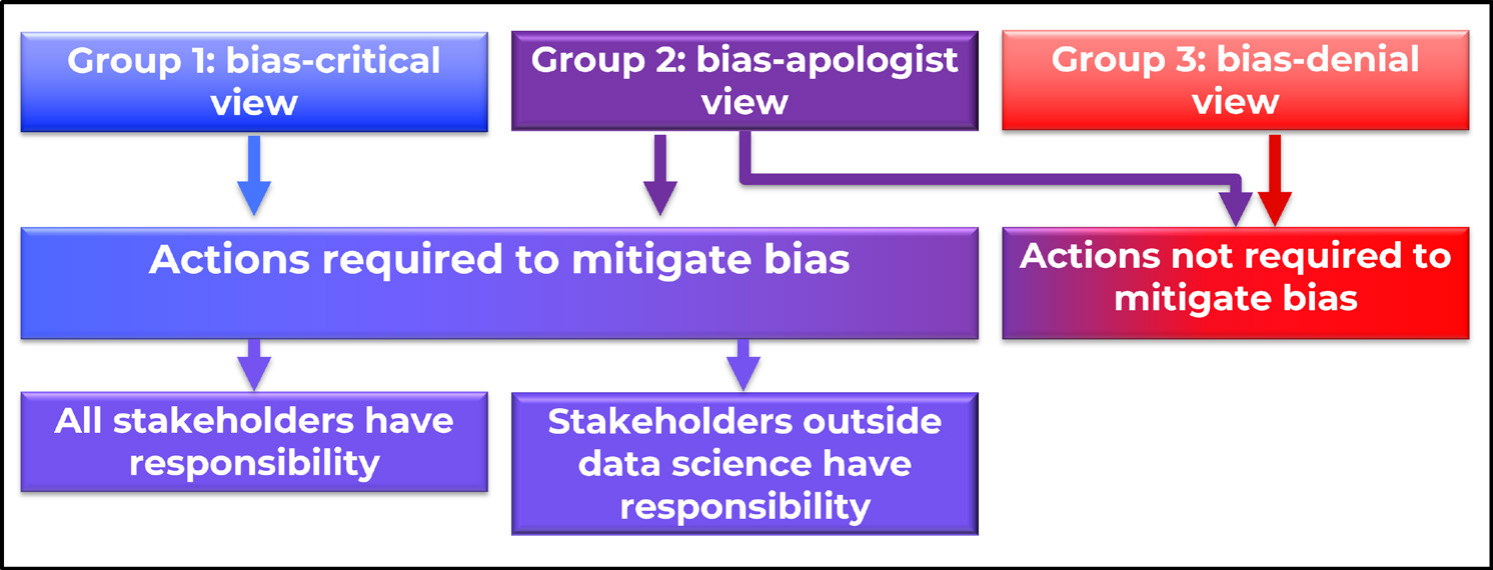
Views about algorithmic bias as a problem.

Participants who expressed the bias-denial view and some who expressed the bias-apologist view implied that no action was required to address bias because according to their views, bias was not a problem in healthcare AI. Some ‘bias-apologist’ participants, mainly developers and entrepreneurs, agreed that bias was a problem but proposed that it was unintentional (see figure 1). Consequently, these developers and entrepreneurs deemed that they were not responsible for the creation of bias, and therefore, held no obligation to redress it. Some ‘bias apologist’ participants who were all entrepreneurs, researchers or clinicians involved in AI development, argued that they were just working with what was available, implying that research and development should not be hampered by concerns about bias.

We’re definitely using biased data sets. So part of my response is that we’re using the data we can get. Informant 23, developer and researcher in bioinformatics

Bias-critical and some bias-apologist participants subscribed to the action-required view, which explicitly calls for action to address the problem of bias. There were two subgroups within this view, distinguished by their views on whose responsibility it is to mitigate bias (see figure 1). In one subgroup, participants, mainly developers and data scientists, maintained that mitigating bias was the responsibility of experts outside science and technology, as illustrated by the statement:

I think people do understand that there is a problem. But as always the question is how do we—I mean from a science point of view the way to address it is to make sure that we have good representation, that we have a wide enough number of samples from all possible classes, or some groups and so on. But how do we ensure that? And that goes outside technology. That goes outside science really … we need more data, and more of the right data. But how we do it I think is outside science and technology really. Informant 51, developer

In contrast, the second subgroup consisting of experts from various roles argued that all professional stakeholders, including data scientists and developers, shared responsibility for and played a role in mitigating AI bias.

So we know about racism, so we expect that we need it to be tested. We know about sexism, so we know it needs to—we know ageism, and we know that that needs [to be tested]. Informant 21, entrepreneur

You’re only as good as the data. And the data is Caucasian predominantly. So—this is another element why human element needs to be in there to, sort, of take the context into account for doing—for assessing the prediction. I think, here, in Australia there’s a special issue with the Indigenous population, for example, where there’s certain diseases that’s much more prevalent in the Indigenous community and it’s just not understood why. Informant 24, researcher in bioinformatics

Both of these two positions—the view that everyone, including data scientists, were responsible, and the view that responsibility lay outside data science—were seen in both bias-critical and bias-apologist informants: there was no observable relationship between these positions.

**Figure 2:**
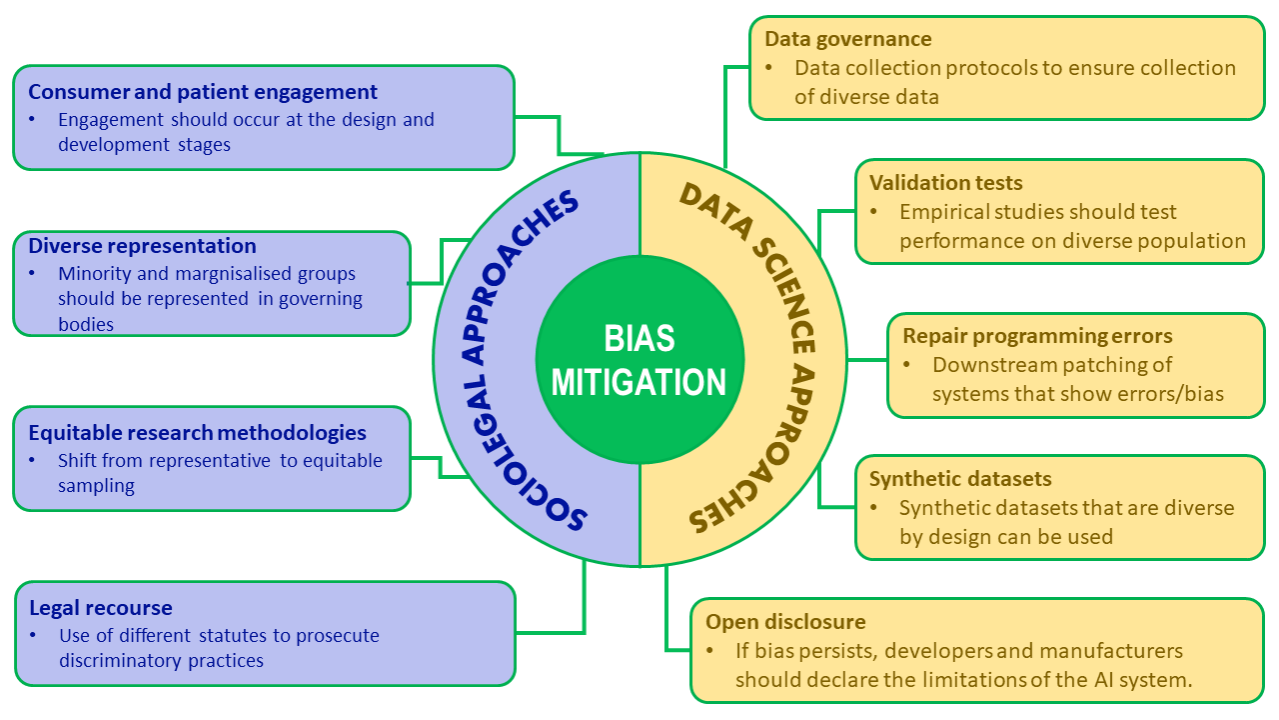
Sociolegal and data science approaches to mitigate bias described by participants.

Those participants who expressed a view that action was required offered mechanisms that fell broadly into two categories, sociolegal approaches and data science mechanisms. Participants who suggested data science mechanisms were predominantly clinicians, developers and researchers. The mechanisms included data governance, validation tests and use of synthetic data sets, among others (see figure 2).

If it remained impossible to remove bias in AI algorithms, some participants recommended open disclosure of limitations: that is, that when communicating about the predictions or recommendations generated by AI, to always openly acknowledge that these were biased or partial.

So hugely important not to have bias, and hugely important to recognise the bias more than anything else … when we publish these trials. We don’t say, this algorithm is great at reading chest x rays, we say this algorithm is great at reading chest x rays in Caucasian males over the age of 70, for example. And for clinicians, we talked a bit before about how much they need to understand about AI. They don’t need to understand anything about AI before they understand everything about statistics. Because how that data is presented, how there are confounders how there is bias, that part is more interesting than the AI. Informant 54, clinician

Other participants, predominantly regulatory and legal experts, discussed sociolegal approaches—in addition to data science mechanisms—to mitigate algorithmic bias. These approaches included consumer and patient engagement, representation of marginalised groups, incorporation of equity considerations into sampling methods, and legal recourse.

You might essentially violate [human rights] if you create an algorithm that produces biased results, so I think—I don’t think—I don’t know if there have been any cases yet, but I think you could not only end up with ethical problem, but also with a legal problem, infringement of human rights, so from legal perspective. So when people talk about bias as an ethical principle, I think they often forget that it’s also a legal issue, so that’s as far as current law is concerned. Informant 32, regulatory expert

**Figure 3:**
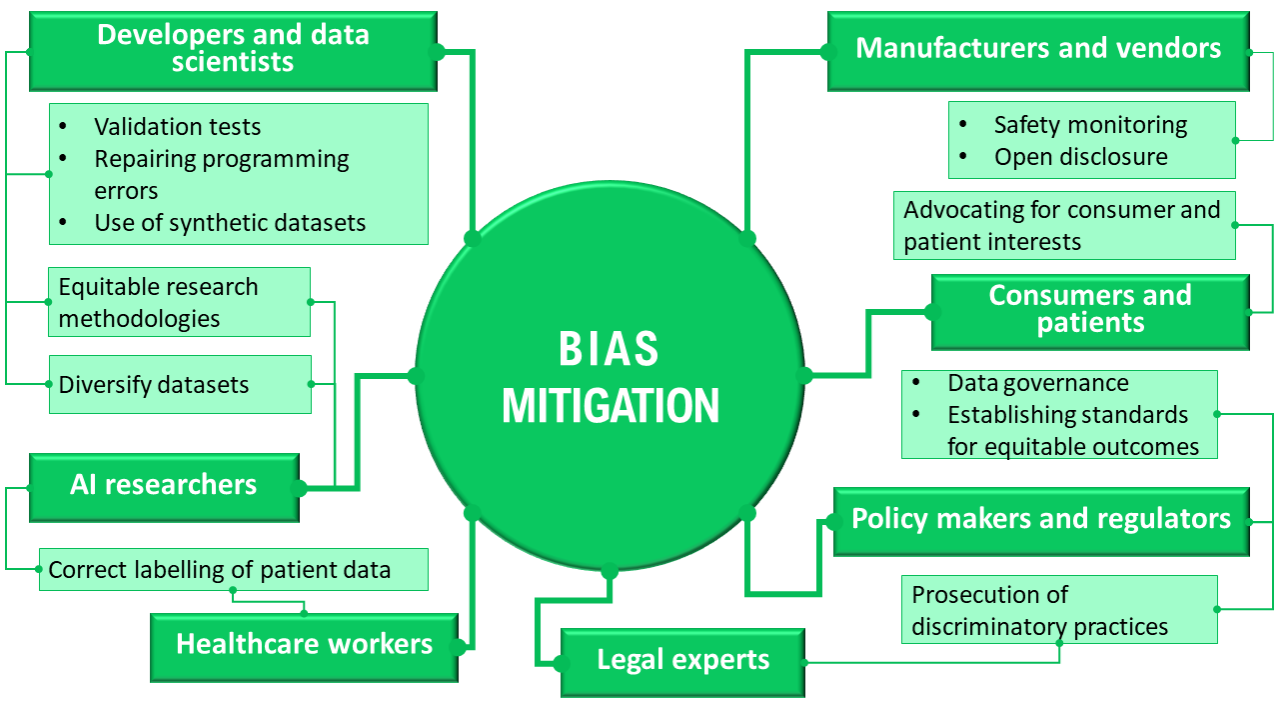
Summary of informants’ perspectives on stakeholder responsibilities regarding bias mitigation.

In addition to describing different approaches to bias mitigation, participants identified a wide range of stakeholders who may be responsible for addressing AI bias, and these included developers, healthcare workers, manufacturers and vendors, policy makers and regulators, AI researchers and consumers. The numerous responsibilities ascribed, which varied in scope, illustrated the complexity of algorithmic bias and potential ways to address it. See figure 3 for a summary. We emphasise that this is a summary of the views expressed by participants rather than our own normative position.

Noting the diversity of possible solutions, as well as the type of stakeholders that should be involved in these solutions, participants identified the need for interdisciplinary collaboration among experts. In particular, an interdisciplinary approach could capture the sociolegal and technical dimensions of the problem and causes of bias, as well as mechanisms to correct causes of and mitigate the impact of bias.

That’s where you need clinicians working with the model developers at the very outset, okay, to say if we’re going to use this tool in a certain way this is—these are the peculiarities of our population, so we need to make sure that we sample the population adequately to make sure that we’re not producing biased data. … So we can’t have I think model developers—model developers by themselves, or data scientists just in isolation. You’ve got to have clinicians and others who have a real understanding of the population that we’re dealing with. Informant 41, clinician

So when we find technical solutions probably we will get next to standards level, to set standards that are [the] minimum standard that each AI application has to meet to … satisfy this fairness criteria and then when we have standards we can then validate—put them in laws and say you will not be liable, let’s say, for breach of discrimination … if you comply with standards in the industry. Informant 32, regulatory and legal expert

### Disagreement 3: views on the role of social identities in addressing bias

One particularly challenging issue when discussing mechanisms to address bias was the role of information regarding complex social identities (eg, race/ethnicity). Participants had divergent views about the role of social data (eg, information about ethnicity or income). A small number of participants, mostly researchers and developers, took an exclusionary view of social data in AI development. This view meant that in order to minimise algorithmic bias, social identities subject to prejudice should be masked or excluded in datasets.

If you are, for example, putting the ethnicity of certain groups as a variable, as a basic variable, and you put much weight of this variable, the whole algorithm will be biased towards or away from these groups. But actually you don’t need to put that because these classifications are human classifications, they are not scientific classifications. Informant 4, developer

Some participants took the inclusionary view of social data in AI development, as well as in addressing algorithmic bias. This heterogeneous group included entrepreneurs, clinicians, consumer representatives and regulatory experts. Several reasons to support the inclusionary view were provided. First, social information could make the dataset more robust and representative.

If you learn through things like race and ethnicities, if it captures employment status, if we don’t have things even such as housing status, sort of include information around sort of behaviours, substance abuse, alcohol abuse, et cetera, that those things aren’t there and EMR [electronic medical records], as we know, sometimes can omit the sort of information unless you have good structured templates where those things are listed and you have to actually include them, okay but we know that often people don’t necessarily provide all that information when they’re admitting a patient or even subsequently. Then, yeah—then that’s right, you’re not going to have a particularly accurate model or that they have a model that’s biased because it doesn’t include those other features. Informant 41, clinician

One of the ones I notice often when I’m looking at surveys or things that ask me about my features, because I’m taking part in a research study, you know, if you look at lists of gender and sexuality, like, asexuality, for example, will be missed from a lot of “pick-an-option” about your sexuality. Right? So, again, people don’t choose it, it doesn’t end up in the system, it doesn’t end up in the coding, it’s more invisible again and it becomes a bit of a cycle, unfortunately. Informant 45, consumer representative

Another reason that supported the inclusionary view was to enable healthcare systems to identify which groups were underserved.

And we also need to know their sociodemographic, and so there is publicly available data that you can connect up but there are people who don’t turn up and they’re the ones that are missing out often. So we need to get the census data, we need to get everything and go, “Well, okay, who are we missing?” So I think we can figure out who’s not connected with our system. They’re either not connected because there’s a reason or they’re well. Informant 31, regulatory expert

Some participants who supported the inclusionary view discussed caveats, such as increased (unjust) surveillance.

I get the argument of yes, there is some truth to that argument, that the more data you have, the more—I mean again, to give you an analogy, you could make similar arguments in the biomedical sphere as well. You could say we would get to cures or medical treatments faster if researchers were allowed to do whatever they wanted in the research phase. Just trial this drug that may kill people on people in prison. That would work. But we don’t do it for really good reasons and that is because we don’t accept that level of harm to innocent people. And that’s true also of, I think, research in this area and again, we’ve talked a bit about facial recognition. We know that facial recognition is much less accurate when it’s used on people with darker skin. And in an area I care deeply about, policing, there’s an argument, okay, we’ll make the training data sets a high proportion of those people with darker skin. But if the outcome of that is that those people will be policed more aggressively, that’s really hard to justify. So I think it’s quite a complex area… You’ve got to actually weigh the costs and benefits and specifically consider the uses to which the application may be put. Informant 37, regulatory expert

A substantial number of participants, mainly developers and regulatory experts, took neither the exclusionary nor the inclusionary view. This uncertain view about the role of social data in AI development took into account the challenges of collecting sensitive and complex information, and translating this into useful data for developing AI algorithms. Data science relies on categories and classifications that are discrete, a feature that is not always possible for social data.

When you say, “What about the culturally and linguistically diverse population?” Right, but for useful purposes you do need information to be much more granular, and that requires people to self-identify and that can be very tricky. I mean, most people don’t simply have one background, they have multiple—I’ve got 10, so what do you use? Informant 10, regulatory expert

We don’t capture all the data we need and even if we could capture it all actually making sense of it all and understanding which of these many, many variables actually matter is really unclear. Informant 38, developer

Thus, the findings show lack of consensus on how to manage, operationalise or capture complex social identities to mitigate algorithmic bias. The divergent views illustrate the tension between ensuring diversity in datasets and the potential harm of collecting data points marked by historical and continuing prejudice or discrimination.

## Discussion

Our aims were to understand whether informants saw algorithmic bias in healthcare as a problem, to capture possible strategies to mitigate algorithmic bias, and to consider the ethical question of responsibility for algorithmic bias. Our answers to these questions have practical, epistemic and normative implications. We note that, as authors, we agree that bias is a concern in AI, including healthcare AI, and that bias mitigation is required. Thus, we do not agree with all of the informants, and the Discussion section will reflect this.

We found that some stakeholders had the view that bias does not exist in healthcare AI, based on the epistemic belief that these knowledge systems are not biased. People taking this view are incorrect as a matter of fact: algorithmic bias has been demonstrated across domains including healthcare,^33^ social welfare,^34^ legal systems^35^ and finance.^36^ In healthcare, studies continue to demonstrate the persistence of racial disparities in diagnostic imaging that predates AI applications. A systematic review of evidence shows disparities in non-AI diagnostic radiology, with decreased and inappropriate use of imaging for racial and gender minority groups.^37 38^ In the USA, members of racial minority groups disproportionately receive care at lower-quality hospitals that use lower-quality imaging technology.^39^ These lower-quality hospitals tend to have limited human and material resources, relying on generalists who are not experienced in diagnostic imaging interpretation.^40^ According to a report by the US Institute of Medicine of the National Academies,^41^ factors including unequal access and unequal quality of care lead increased risk of diagnostic error among racial minority groups.^39^ Similar patterns of disparities in quality or delivery of diagnostic imaging have been shown across various multi-cultural jurisdictions including Australia^42^ and the UK.^43^ The entrenched disparities in diagnostic imaging skew the availability and quality of data on which AI is trained, and thus perpetuating the data imbalance that contributes to algorithmic bias. Moreover, disparities in access to healthcare services, not just in diagnostic radiology, can be embedded in datasets used to develop algorithms. Consider, for example, a commercial algorithm widely used in the USA, designed to direct additional healthcare resources to patients at high risk of exacerbation or complications of their illness. Obermeyer *et al*^16^ showed that this algorithm, which was trained on datasets that did not contain information about race or ethnicity, systematically underenrolled black patients, even if they were sicker than their white counterparts. Obermeyer’s team showed that the algorithm used health costs, which stratify populations along racial lines,^44^ as proxy for health needs. Since less money is generally spent on black patients due to existing systematic bias, the algorithm predicted that black patients had less need for high-risk management.^16^

For other participants who denied the existence of algorithmic bias, the denial concerned the relevance of the issue rather than the absence of bias itself, holding that these knowledge systems are biased, but this is normatively acceptable. Either of these views (denial or minimisation) present a barrier to action: these beliefs make responsibility to address bias non-existent and actions to address bias unnecessary. The only position that supported action to mitigate bias was the bias-critical view. It seems likely that those who are bias-critical in professions including data science and medicine will need to convince their colleagues if bias is to be effectively mitigated. As bias produces unjust outcomes, claiming that bias does not exist and does not require mitigation is not normatively defensible. Working to effectively counter these views will require recognition that bias is often implicit.^44^

This returns us to the issue of responsibility for addressing the complex problem of algorithmic bias.^45^ In disciplines including law, ethics and political philosophy, the concept of responsibility is used in varied ways.^2446^,^48^ Earlier we introduced Fricker’s distinction between fault and no-fault types of responsibility in the context of prejudice,^24^ and her argument that we are rightly held responsible ‘not only for conduct based on things we know but also for conduct based on things we should have known but didn’t’. One might expect that experts who are well-informed about AI are similarly well informed about the risk of bias. Our study, which included a small group of experts who denied the existence of bias do so because they have unknowingly absorbed norms and prejudices from society. However, following Fricker, we argue they should have known, and so still have responsibility for algorithmic bias and its effects. Those who acknowledge the existence of bias but are apologists for it may be making a moral judgement rather than an epistemic one. They seem to imply that they are not responsible for action because they are not in control in a relevant sense: however, as Fricker argues, people can be held responsible not just for those things they control, but also those things they ‘ought to be able to control’. This connects to participants’ proposals about bias mitigation.

When participants proposed mitigation strategies, ranging from sociological to data science approaches (see figure 3 above), they allocated responsibility to implement those strategies to different parties. For some participants, responsibility for addressing bias lies outside science and technology. One possible interpretation of this view is that experts in science and technology are not at fault for structural and systemic biases that are then reflected in AI bias, and therefore, should not be responsible for addressing the problem. Another interpretation is that experts deem that structural and systemic factors that lead to bias are outside their training and expertise, and they feel ill equipped to address these factors. Both interpretations, but perhaps the first more than the second, require a normative response.

Drawing on Fricker’s notion of no-fault responsibility, responsibility to mitigate bias need not be based on fault, attributing blame or contributing to cause. In as much as there are aspects of their own professional domain that they ought to be able to control, they are responsible even if they did not cause the problem. The complex, systemic and multisectoral problem of bias seems likely to require an equally complex, systemic and multisectoral response. The mechanisms required to address bias are located in different disciplines, each offering distinct knowledge and skills. In a practical sense, if all these strategies are required, it follows that an interdisciplinary collaboration among diverse experts will be required to effectively address bias. It seems likely this will require mutual engagement between technical AI experts and those with expertise in the socio-political dimensions of injustice in healthcare, which in turn indicates that that appeals to feeling ill equipped or lacking in expertise will fall normatively short.

In relation to specific mitigation strategies, we note participant disagreement about the handling of data concerning complex social identities, such as gender and racial identities. It is difficult to capture information that requires self-identification, that does not adhere to discrete categories, and that is subject to prejudice and marginalisation. In an epistemic sense, participants said they lacked hermeneutic resources to represent complex social identities in data. This lack of hermeneutic resources is one outcome—as pointed out by critics^49^—of the AI research and development workforce being overwhelming white and male. This means standards of practice are dictated by those in positions of privilege who may not fully understand the reality—including the causes and impact—of algorithmic bias. However, many of the sociolegal strategies advocated by participants were designed to increase the availability of hermeneutic resources, including engagement, representation and equitable research methodologies. Normatively speaking, it is not clear which of these strategies should be prioritised and pursued, given that there is currently no concrete guidance about engaging with or managing data points for complex social identities. Further research is needed to demonstrate the extent to which this lack of guidance on managing data on social identities is contributing to algorithmic bias. These findings echo ongoing calls for ‘open science’,^14^ which refers to a framework where the entire research process is openly shared to incentivise participation of different stakeholders, as well as encourage members of vulnerable or under-represented groups to become part of the global scientific community.

The strength of this study is identifying divergence in the views of a diverse group of experts involved in the development, acquisition, deployment and regulation of healthcare AI regarding the problem of algorithmic bias. Even if it is not clear which strategies to implement and whose responsibility it is to implement those strategies, our findings offer some practical suggestions canvassed from experts. However, our study has limitations. First, the topics covered in each interview were wide-ranging, and may at times have been at the expense of depth of discussion. Second, we did not collect data on gender and racial identities, as our focus was on understanding differences between different professional groups. We were thus unable to investigate any links between participant demographics and critical, apologist or denialist viewpoints. Future research could use stratified sampling to ensure representation of different racial and gender identity groups, and to enable analysis of relationships between demographics and views about bias. Third, while we made all efforts to clarify concepts such as bias and prejudice during the interviews, these concepts were too complex and participants had different interpretations or definitions depending on their expertise.

## Conclusion

AI applications can improve healthcare services and bring benefits to society. However, there is a growing evidence that benefits may not be equitably distributed due to AI replicating or amplifying existing social biases in healthcare that continue to disadvantage already marginalised and underserved individuals or groups. Despite the growing evidence of AI bias, our qualitative study of expert perspectives shows disagreements about (1) whether bias exists, (2) what actions to mitigate bias are needed and (3) how to handle information about complex identities such as gender and racial identities. We argue that these disagreements provide valuable information about barriers to bias mitigation. In particular, we highlighted the interrelated practical, epistemic and normative implications of participants’ views. We argue that stakeholders are responsible for addressing bias in algorithmic systems, even when they deny its existence, or claim they are not responsible for acting. Actions to start addressing bias include greater and earlier interdisciplinary collaboration from AI development through testing and application, tailored stakeholder engagement activities, empirical studies to understand algorithmic bias, and strategies to modify dominant approaches in AI development such as use of participatory methods, and increased diversity and inclusion in research teams and research participant recruitment and selection.

## Footnotes

In our discussions with expert participants, they did not necessarily adhere to formal definitions of bias, but rather reflected the common-sense usage of “bias” that tends to capture both the statistical meaning (i.e. technical error) and social meanings (negative attitudes and discriminatory practices).

## Data availability statement

Data sharing not applicable as no datasets generated and/or analysed for this study.
